# Efficacy and Tolerability of Peginterferon **α**-2a and Peginterferon **α**-2b, Both plus Ribavirin, for Chronic Hepatitis C: A Meta-Analysis of Randomized Controlled Trials

**DOI:** 10.1155/2013/739029

**Published:** 2013-04-11

**Authors:** Zongguo Yang, Liping Zhuang, Lei Yang, Xiaorong Chen

**Affiliations:** ^1^Shanghai Public Health Clinical Center Affiliated to Fudan University, Department of Traditional Chinese Medicine, No. 2901 Caolang Road, Jinshan District, Shanghai 201508, China; ^2^Shanghai Medical College, Fudan University, Department of Oncology, Shanghai 200032, China; ^3^Shanghai Cancer Center, Department of Integrative Medicine, Shanghai 200032, China; ^4^The Central Hospital of China Aerospace Corporation, Beijing 100049, China

## Abstract

*Background*. The efficacy and tolerability of peginterferon **α**-2a and peginterferon **α**-2b in chronic hepatitis C (CHC) patients remain controversial. *Methods*. PubMed, Ovid, and Cochrane libraries were electronically searched until August 30, 2012. Studies that met the inclusion criteria were systematically evaluated by two reviewers independently. *Results*. The overall sustained virologic response (SVR) rate of the peginterferon **α**-2a group was significantly higher than that of the peginterferon **α**-2b group (46.7% versus 42.4%, *P* value = 0.01). The same tendency was observed for naïve, genotype 1/4, and genotype 2/3 patients. The early virologic response (EVR) and end-of-treatment response (ETR) rates were significantly higher in the peginterferon **α**-2a group than in the peginterferon **α**-2b group (56.1% versus 49.8%, *P* < 0.0001; 67.9% versus 56.6%, *P* < 0.00001, resp.). Peginterferon **α**-2a had a significantly lower discontinuation rate than peginterferon **α**-2b (27.9% versus 33.9%, *P* < 0.0001) in naïve patients. In both naïve CHC and hepatitis C virus genotype 1 patients, peginterferon **α**-2a had a higher relapse rate than peginterferon **α**-2b. *Conclusions*. Peginterferon **α**-2a has superior efficacy with higher EVR, ETR, and SVR than peginterferon **α**-2b for CHC patients, both plus ribavirin. Peginterferon **α**-2a might obtain a similar or even lower discontinuation rate than peginterferon **α**-2b. However, peginterferon **α**-2a had a higher relapse rate than peginterferon **α**-2b.

## 1. Introduction

The World Health Organization has estimated that up to 170 million people (approximately 3% of the world population) worldwide might be infected with hepatitis C virus (HCV). This virus is responsible for approximately 350,000 deaths every year. HCV is cleared spontaneously in only approximately 20% of individuals. Chronic infection frequently progresses to cirrhosis, end-stage liver disease, hepatocellular carcinoma, and death [1–4].

Currently, in many countries, the recommended therapy for chronic hepatitis C (CHC) is still the combination of peginterferon *α* and ribavirin [1, 2]. Two licensed products of peginterferon *α* are available: peginterferon *α*-2a (Pegasys, Hoffmann-La Roche, Nutley, NJ, USA) and peginterferon *α*-2b (Peg-Intron, Schering Plough Corp., Kenilworth, NJ, USA). However, differences in structural modifications and dosing (weight-adjusted versus fixed) between the two peginterferons may lead to various clinical outcomes. In addition, a recommendation about the two regimens has not been proposed in the current guidelines [5–11]. Although recent studies have compared the response rates obtained using the two peginterferons in CHC, they have failed to reach a consensus as to which treatment options are the most effective.

Some systematic reviews [12–15], which include meeting abstracts or HCV/HIV coinfected patients, concluded that peginterferon *α*-2a has higher sustained virologic response (SVR) than peginterferon *α*-2b in CHC but revealed that both have similar safety. The virologic responses and tolerability of peginterferon plus ribavirin in HCV/HIV coinfected patients are substantially different from those in chronic HCV monoinfected patient. In addition, some reported meeting abstracts were found to be inadequate. Thus, we performed a meta-analysis of randomized controlled trials (RCTs) with critical inclusion and exclusion criteria to evaluate the efficacy and tolerability of the two regimens.

## 2. Materials and Methods

### 2.1. Search Strategy

We searched PubMed, Ovid, and Cochrane libraries until August 30, 2012. The following medical subject headings were used: “Hepatitis C, Chronic,” “interferons,” “peginterferon alfa/alpha/*α*-2a,” “peginterferon alfa/alpha/*α*-2b,” and “ribavirin.” Electronic searches were supplemented with manual searches of reference lists of all retrieved review articles, primary studies, and abstracts from meetings to identify other studies not found in the electronic searches. The literature was searched by two authors (Z. Yang and L. Zhuang) independently.

### 2.2. Study Selection

Two authors independently selected trials and discussed them with each other when inconsistencies were found. Articles that meet the following criteria were included: (1) study types, randomized controlled trials; (2) participants, chronic HCV virus monoinfection patients either naïve or retreatment were randomly divided into two groups; (3) interventions, peginterferon *α*-2a and peginterferon *α*-2b, both plus ribavirin; (4) outcome measures, studies that used one or more of the following measurements were eligible: rapid virologic response (RVR), early virologic response (EVR), end-of-treatment virologic response (ETR), SVR, relapse rate, and discontinuation rate; and (5) full texts available.

Studies with the following situations were excluded: (1) followup period less than 6 months and (2) studies that included patients with other liver diseases (e.g., HBV infection, human immunodeficiency virus infection, and hepatocellular carcinoma) aside from HCV.

### 2.3. Quality Assessment

The methodological qualities of the included RCTs were assessed according to Cochrane Collaboration's tool described in Handbook version 5.1.0 [16]. Two authors (Z. G. Yang and L. Yang) assessed the quality independently, and inconsistency was discussed with a third review author (X. R. Chen) who acted as an arbiter.

### 2.4. Data Extraction

Two researchers read the full texts independently and extracted the following contents: publication data (first author's name, year of publication, and country of population studied), study design, sample size, patient characteristics (age, gender, body weight, distribution of genotype, and liver histology), treatment protocol (peginterferon type and dose, ribavirin dose), outcome measures (RVR, EVR, ETR, SVR, relapse rate, and discontinuation rate), and reasons for discontinuing combination therapy. Authors were contacted by e-mail for additional information if data were unavailable.

### 2.5. Definitions

Chronic hepatitis C is defined by anti-HCV positive, HCV RNA positive as determined by a qualitative polymerase chain reaction (PCR) assay for more than 6 months. The primary outcome measure of efficacy of SVR was defined by a sensitive PCR assay as the absence of HCV RNA from serum at 24 weeks after completion of therapy. Secondary outcome measures of tolerability, including discontinuation rate, RVR, EVR, and ETR, were also determined. RVR was defined using a sensitive PCR assay as undetectable HCV RNA at 4 weeks after treatment. EVR was defined as ≥2 log reduction or complete absence of HCV RNA at 12 weeks after therapy compared with the baseline level. Undetectable virus at the end of either a 24-week or 48-week course of therapy was referred to as ETR. Virologic relapse refers to the reappearance of HCV RNA in serum after treatment was discontinued and ETR was documented.

### 2.6. Statistical Methods

Data were processed in accordance with the Cochrane Handbook [16]. Intervention effects were expressed with odds ratios (ORs) and associated 95% confidence intervals (CIs) for dichotomous data. By contrast, the effects were expressed with mean differences and 95% CIs for continuous data. Heterogeneity among studies was informally assessed by visual inspection of forest plots and formally estimated using *χ* ^2^ and *I* ^2^ tests (both *P* > 0.05; *I* ^2^ < 50% indicates no evidence of heterogeneity between the pooled studies) [17]. The fixed-effects model was first used for meta-analyses. The random-effects model was used in the presence of heterogeneity. Description analysis was performed when the quantitative data could not be pooled. Intention-to-treat (ITT) principle was used. Review Manage (v. 5.1; The Cochrane Collaboration) was used for data analysis.

## 3. Results

### 3.1. Study and Patient Characteristics

 A total of 1166 abstracts of clinical trials were found and reviewed. Of these 1166 abstracts, 45 were retrieved, 6 [18–23] were excluded because they were published as abstract proceedings, 1 [24] was excluded because patients received monotherapy of peginterferon *α*-2a/2b at the first 4 weeks, 1 [25] was excluded because it was not designed randomly, 1 [26] was excluded because patients received 1.0 *μ*g/kg peginterferon *α*-2b, 1 [27] was excluded because it included patients with HCV/HIV coinfection, and 1 [28] was excluded because duplicate data from the same medical center were published. Finally, 7 trials [5–11] met our inclusion criteria (Table 1).

 Totally 1845 and 1823 patients were randomly treated with peginterferon *α*-2a and peginterferon *α*-2b, respectively, both plus ribavirin. The baseline characteristics of each study included in this meta-analysis are described in Table 2.

### 3.2. Methodological Quality Assessment

**Figure 1:**
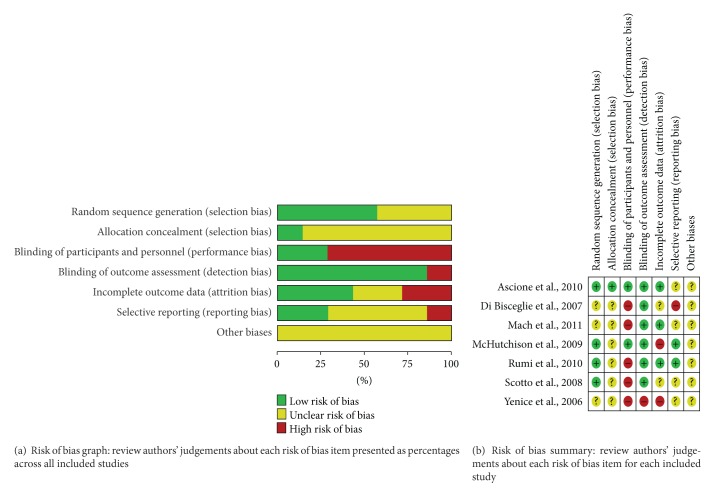
Risk of bias assessment.

All studies included in this meta-analysis were described as randomized. Three studies [5, 6, 11] did not report the method of randomization, but randomization was adequate in other studies [7–10]. Among these studies, two were randomized by a computer-generated randomization list [9, 10], one was randomized by an interactive voice system [8], and the study by Scotto et al. was randomized by a table of random numbers [7]. One study revealed that the randomization list was not available to the treating physicians. Double blinding was described in one trial by McHutchison et al. [8]. And, Ascione et al. [10] designed a study where the physician received the report on the allocation of each patient from an independent researcher who knew nothing about the patient except for the genotype. The statistical analyses in one study by Yenice et al. [5] were not based on ITT, and more than 20% of the participants in the study by McHutchison et al. were lost to followup, both of which were considered as high risk in the item of incomplete outcome data. No descriptions of lost to followup were found in the two studies by Di Bisceglie et al. [6] and Scotto et al. [7], thus accounting for the ambiguity in the item of incomplete outcome. No patient was lost to followup in the study by Ascione et al., and the other studies described the lost to followup participants, which were balanced between the two groups and considered low risk. Selective reporting was found in the study by Di Bisceglie et al. because it failed to include the expected results (e.g., SVR rate) for such a study. The other potential biases were unclear in these trials (Figure 1). 

### 3.3. Virologic Responses

**Figure 2:**
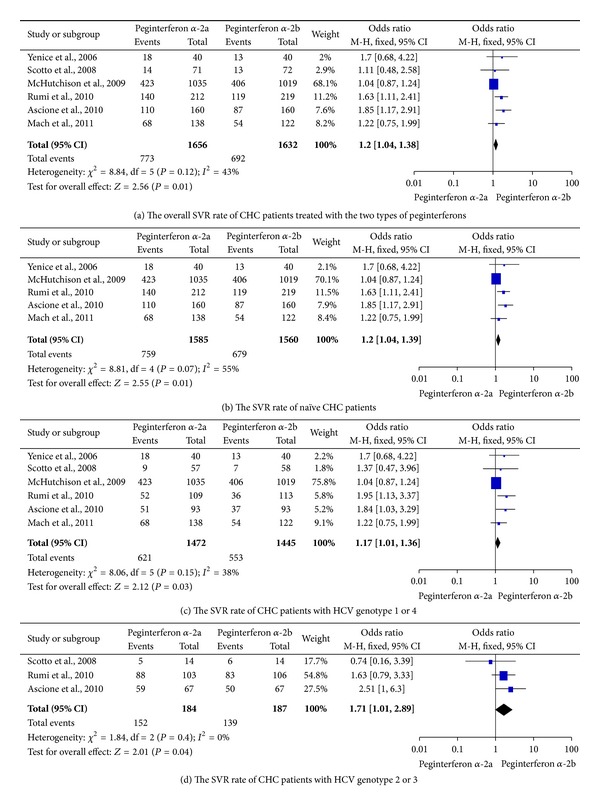
SVR rates of chronic hepatitis C patients who received the two regimens of peginterferon *α*-2a and peginterferon *α*-2b, both plus ribavirin.

The overall SVR rates for CHC patients treated with peginterferon *α*-2a plus ribavirin and CHC patients treated with peginterferon *α*-2b plus ribavirin were 46.7% (773/1656), and 42.4% (692/1632), respectively (OR = 1.20, 95% CI = 1.04–1.38, and *P* = 0.01; Figure 2(a)). For naïve patients with no interferon experience, subgroup analysis found that the SVR rate was significantly higher in the peginterferon *α*-2a group than in the peginterferon *α*-2b group (47.9% versus 43.5%, OR = 1.20, 95% CI = 1.04–1.39, *P* = 0.01, Figure 2(b)). For genotype 1/4 patients, peginterferon *α*-2a could obtain a higher SVR than peginterferon *α*-2b (42.2% versus 38.3%, OR = 1.17, 95% CI = 1.01–1.36, *P* = 0.03, Figure 2(c)). For CHC patients with genotype 2/3, peginterferon *α*-2a might achieve a higher SVR rate than peginterferon *α*-2b (82.6% versus 74.3%, OR = 1.71, 95% CI = 1.01–2.89, and *P* = 0.04; Figure 2(d)).

**Figure 3:**
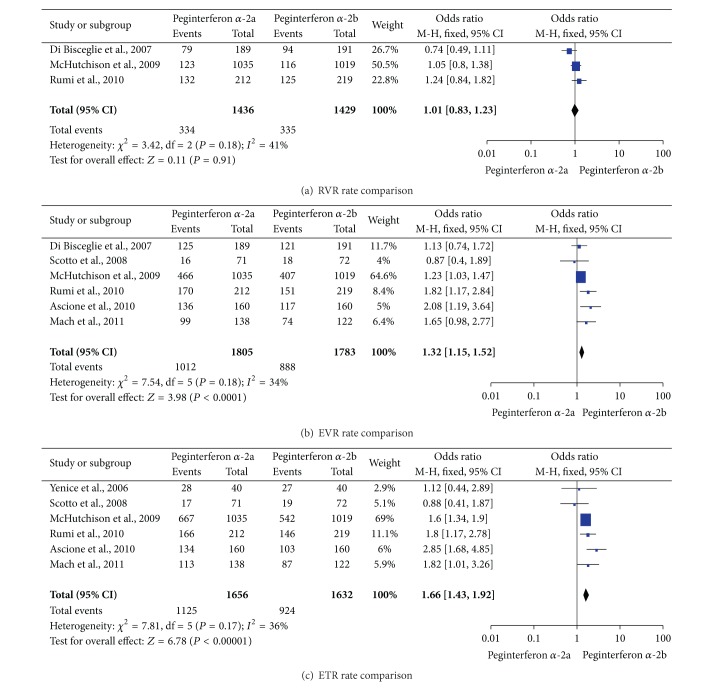
The RVR, EVR, and ETR rates of CHC patients treated with the two regimens.

Only three studies [6, 8, 9] reported the RVR rate in patients who received peginterferons plus ribavirin. No difference in RVR rate was found between the two regimens (23.2% versus 23.4%, OR = 1.01, 95% CI = 0.83–1.23, and *P* = 0.91; Figure 3(a)). However, patients treated with peginterferon *α*-2a could achieve significantly higher EVR rates than those treated with peginterferon *α*-2b (56.1% versus 49.8%, OR = 1.32, 95% CI = 1.15–1.52, and *P* < 0.0001; Figure 3(b)). Meta-analysis of RCTs [5, 7–11] by a fixed-effects model (*P* = 0.17, *I* ^2^ = 36%) revealed that, compared with peginterferon *α*-2b, peginterferon *α*-2a increased the ETR rate significantly in patients with chronic hepatitis C (67.9% versus 56.6%, OR = 1.66, 95% CI = 1.43–1.92, and *P* < 0.00001; Figure 3(c)).

### 3.4. Discontinuation Rate and Dose Modification

**Figure 4:**
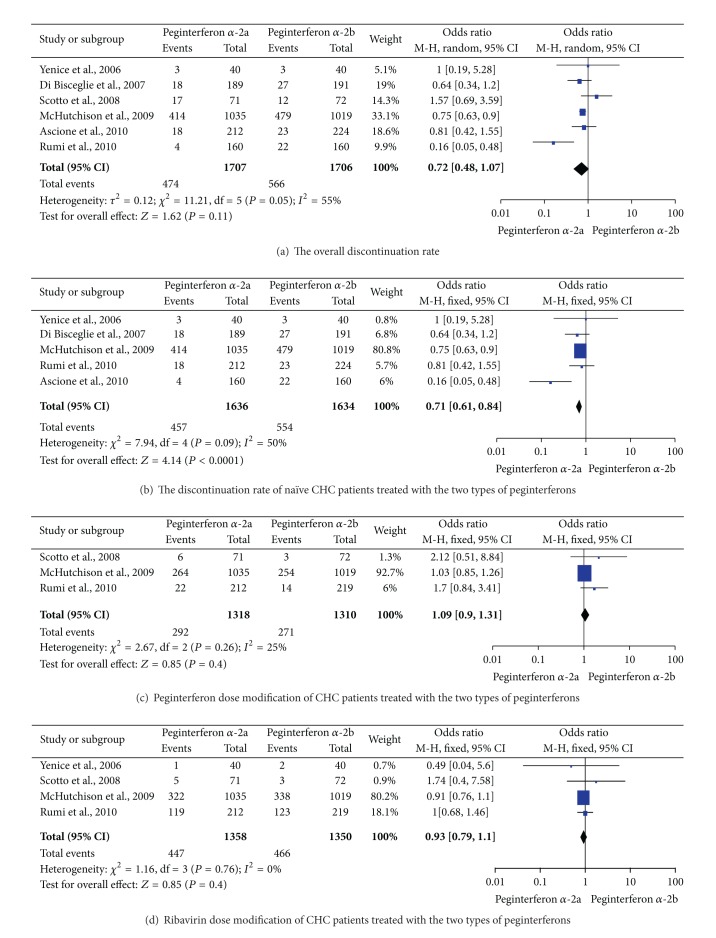
The discontinuation rates and drugs modification of CHC patients who received the two regimens.

All the patients that did not complete the treatment duration were considered as discontinuing therapy, either for adverse events or nonsafety reasons. Of the studies included in this meta-analysis, two [6, 7] reported the number of patients who withdrew from therapy for nonsafety reasons, whereas one [11] did not provide the exact discontinuation number of patients. Meta-analysis of RCTs [5–10] by a random-effects model (*P* = 0.05, *I* ^2^ = 55%) revealed that peginterferon *α*-2a and peginterferon *α*-2b had a similar discontinuation rate for CHC patients, including naïve and retreatment ones with any HCV genotype (*P* = 0.11, Figure 4(a)). By contrast, meta-analysis of RCTs [5, 6, 8–10] by a fixed-effects model (*P* = 0.09, *I* ^2^ = 50%) revealed that peginterferon *α*-2a had a significantly lower discontinuation rate than peginterferon *α*-2b for naïve CHC patients (27.9% versus 33.9%, OR = 0.71, 95% CI = 0.61–0.84, and *P* < 0.0001; Figure 4(b)).

No adequate data of peginterferon *α* or ribavirin dose reduction were reported in the studies by Yenice et al. [5], Di Bisceglie et al. [6], Ascione et al. [10], and Mach et al. [11]. However, the same dose reduction was applied for both arms in two studies [6, 10]. For the modification of peginterferon dose, meta-analysis of RCTs [7–9] by a fixed-effects model (*P* = 0.26, *I* ^2^ = 25%) indicated no difference in the two types of peginterferons (22.2% versus 20.7%, OR = 1.09, 95% CI = 0.90–1.31, and *P* = 0.40; Figure 4(c)). For the reduction of ribavirin dose, meta-analysis of RCTs [5, 7–9] by a fixed-effects model (*P* = 0.76, *I* ^2^ = 0%) revealed no statistical difference between the two groups (32.9% versus 34.5%, OR = 0.93, 95% CI = 0.79–1.10, and *P* = 0.40; Figure 4(d)).

### 3.5. Relapse Rate

**Figure 5:**
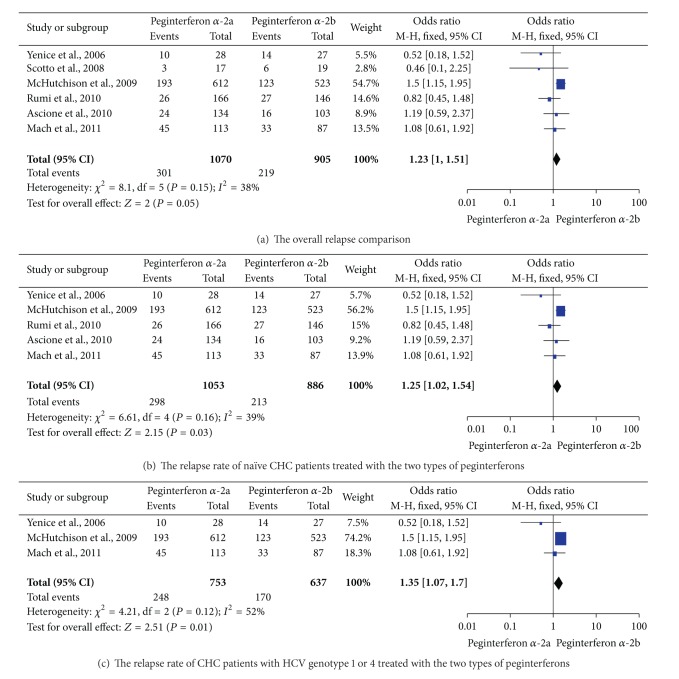
The relapse rate of CHC patients who received the two regimens.

No difference in relapse rate for CHC patients treated with the two regimens was noted in the meta-analysis of RCTs [5, 7–11] by a fixed-effects model (28.1% versus 24.2%, OR = 1.23, 95% CI = 1.00–1.51, and *P* = 0.05; Figure 5(a)). However, subgroup analysis showed that, for naïve CHC patients, peginterferon *α*-2a obtained a higher relapse rate than peginterferon *α*-2b (28.3% versus 24.0%, OR = 1.25, 95% CI = 1.02–1.54, and *P* = 0.03; Figure 5(b)). For HCV genotype 1 patients, peginterferon *α*-2a had a higher relapse rate than peginterferon *α*-2b (32.9% versus 26.7%, OR = 1.35, 95% CI = 1.07–1.70, and *P* = 0.01; Figure 5(c)).

## 4. Discussion

Most previous meta-analyses concluded that peginterferon *α*-2a has higher SVR rate than peginterferon *α*-2b in CHC patients, but no difference in the safety profile was noted [12–15]. However, a recent meta-analysis has revealed that these two types of peginterferons have similar effects on RVR, SVR, and tolerability [29]. Moreover, the above analyses included either meeting abstracts or coinfected patients of HIV/HCV, which may have an impact on the conclusions. In the present meta-analysis, we included more RCTs and restricted our trial analyses to full papers. We excluded abstracts because they did not contain adequate details of patients and outcomes. 

Interferon-based therapy could lower the risk of cirrhosis and hepatocellular carcinoma and improve the survival of CHC patients who have an SVR with a large possibility through eradicating HCV and cutting liver fibrosis procession. Our analysis showed that peginterferon *α*-2a might achieve a higher SVR rate than peginterferon *α*-2b, including nonresponders. Subgroup analysis revealed that peginterferon *α*-2a was also more effective than peginterferon *α*-2b for HCV genotype 1 or 4 patients or treatment-naïve patients. However, these two types of peginterferons had similar SVR effects on HCV genotype 2 or 3 patients. These analyses indicated a difference in antiviral activity between the two therapeutic regimens. A previous study [30] proved that combination therapy with peginterferon *α*-2a is an independent pretreatment predictor of SVR (OR = 1.88, 95% CI = 1.20–2.96). Peginterferon *α*-2a achieves higher SVR rates than peginterferon *α*-2b in patients infected with HCV-1 and HCV-2; however, the two therapeutic regimens obtain similar SVR rates in patients infected with HCV-3 and HCV-4 [9]. Our results indicated that patients with genotype 2 or 3 had similar SVR rates in both groups. Given that the patients included in this meta-analysis mostly had HCV genotype 1 or 4, only less than 200 patients in each group were infected with HCV genotype 2 or 3; high-quality trials with a large sample size are needed to estimate the efficacy of the two regimens for genotype 2 or 3 CHC patients, especially for the comparison of the therapeutic efficacy in each genotype stratum.

Further analysis showed that no significant difference in RVR rate was found in the patients treated with the two peginterferon-*α*-based regimens. However, peginterferon *α*-2a could achieve higher EVR and ETR rates in CHC patients than peginterferon *α*-2b. Early eradication of HCV is important to the therapeutic resolution of CHC, and RVR remains the most notable on-treatment response predictor of SVR. Moreover, the present guidelines concluded that the absence of EVR is the most robust means of identifying nonresponders. Approximately 97%–100% of the treatment-naïve patients with HCV genotype 1 infection who did not reach EVR failed to elicit SVR. Thus, patients without EVR can discontinue therapy early without compromising their chance to elicit SVR [1, 2]. This finding might be associated with the potentially higher SVR rate of patients treated with peginterferon *α*-2a. ETR does not accurately predict the occurrence of SVR; however, ETR is necessary for SVR to take place [1, 2, 31].

Our meta-analysis of RCTs [5–10] suggests that the two peginterferons may be comparable with regard to any reasons leading to treatment discontinuation, including naïve and retreatment patients with any HCV genotype. However, for naïve CHC patients, peginterferon *α*-2a had a significantly lower discontinuation rate than peginterferon *α*-2b. Previous meta-analyses [12–15] concluded that peginterferon *α*-2a has a similar safety profile as peginterferon *α*-2b. Given that our results were based on ITT analysis, all patients who withdrew therapy were considered as treatment discontinuation, either for adverse events or nonsafety reasons. The reason above may explain why our analysis of discontinuation rate in naïve CHC patients conflicted with those of the previous studies.

Although peginterferon *α*-2a should achieve higher virologic responses and gain lower discontinuation rate, peginterferon *α*-2a had a higher relapse rate than peginterferon *α*-2b. The high relapse rate with peginterferon *α*-2a was a novelty, as in previous studies. Relapse rates ranged from 17% to 25% for peginterferon *α*-2a in patients with HCV genotype 1 [32, 33], which is significantly lower than the 31.5% reported in the IDEAL study [8]. These findings were not supported by two randomized studies that reported no difference in relapse rate between the two regimens [9, 10]. Many factors might have contributed to the difference in the findings above. Some of these factors include differences in epidemiological and genetic characteristics, mean body weight, distribution of genotype CC in the IL28B polymorphism, and ribavirin dose reduction schemes applied to the two regimens [34]. Maintaining a high ribavirin dose (≥12 mg/kg/day) during the full treatment period can lead to suppression of relapse in HCV-1 patients responding to peginterferon *α*-2b plus ribavirin. Ribavirin dosing seems to be instrumental in preventing posttreatment relapse [35], and ribavirin concentration in the later stages of treatment is an important marker for discriminating relapse [34, 36]. In the present meta-analysis, no significant difference in peginterferon and/or ribavirin dose reduction was found between the two groups. However, in the IDEAL study by McHutchison et al. [8], the dose reduction for the peginterferon *α*-2b arm occurred in two steps. The first step was a reduction of either 200 mg (in patients receiving 800 mg/day–1,200 mg/day of ribavirin) or 400 mg (in patients receiving 1,400 mg/day). The second step was reduction by another 200 mg, if required for resolution of the adverse event. For the peginterferon *α*-2a arm, the dose was reduced to 600 mg/day. The abrupt reduction of ribavirin dose to 600 mg/day might have played a crucial role in the high relapse rates observed in patients receiving the peginterferon *α*-2a regimen [8–10, 34].

Therefore, the peginterferon *α*-2a regimen holds a slight advantage in terms of virologic responses and discontinuation rates compared with the peginterferon *α*-2b regimen. This advantage may be considered as a direct consequence of the better pharmacokinetic profile of peginterferon *α*-2a than peginterferon *α*-2b. The pharmacodynamic properties of peginterferon *α*-2a allow slower absorption and elimination than peginterferon *α*-2b. Therefore, maximum concentrations occur later with peginterferon *α*-2a than with peginterferon *α*-2b. Peginterferon *α*-2b is associated with fluctuating blood levels and rapid rise and fall in the blood level because of the relatively rapid release of interferon *α*-2b molecule [37–39]. Previous studies [38, 40] showed that the concentration of peginterferon *α*-2b did not remain stable over the week as a whole. At the end of the week, serum interferon could not be detected in most patients treated with peginterferon *α*-2b. When interferon was no longer detectable in the serum, the viral load increased until the next interferon injection. This phenomenon increases the potential for more side effects and reduces the efficacy of the drug. Peginterferon *α*-2b is distributed widely in the body fluids and tissues [14, 39]. By contrast, peginterferon *α*-2a is distributed predominantly to the blood and interstitial fluid, resulting in high drug concentrations in the liver. The reduced clearance of peginterferon *α*-2a, as a consequence of metabolism via nonspecific proteases, provides significant, consistent, and measurable therapeutic plasma levels even at the end of the weekly dosing period [41]. These differences between the two types of peginterferons should lead to better compliance and superior safety of peginterferon *α*-2a [14].

In conclusion, current evidence suggests that peginterferon *α*-2a has superior efficacy with higher EVR, ETR, and SVR than peginterferon *α*-2b for CHC patients, both plus ribavirin. Peginterferon *α*-2a might obtain similar or even lower discontinuation rate than peginterferon *α*-2b. However, peginterferon *α*-2a had a higher relapse rate than peginterferon *α*-2b. Further trials must focus on the comparison of the two types of peginterferons in terms of achieving SVR and clinically relevant outcomes, such as liver-related cirrhosis, hepatocellular carcinoma, mortality, and morbidity.

## Figures and Tables

**Table 1:** Baseline characteristics of the included trials in this meta-analysis.

Study	Peginterferon	Ribavirin	Baseline treatment history	HCV genotype	Treatment in weeks	Country	Publication year	Study type
Yenice et al. [5]	*α*-2a 180 ug/week; *α*-2b 1.5 ug/kg/week	800–1200 mg/day	Naïve	1	24 or 48	Turkey	2006	RCT
Di Bisceglie et al. [6]	*α*-2a 180 ug/week; *α*-2b 1.5 ug/kg/week	1000–1200 mg/day	Naïve	1	12	USA	2007	RCT
Scotto et al. [7]	*α*-2a 180 ug/week; *α*-2b 1.5 ug/kg/week	15 mg/kg/day	Nonresponders	1,2,3,4	48	Italy	2008	RCT
McHutchison et al. [8]	*α*-2a 180 ug/week; *α*-2b 1.5 ug/kg/week	800–1400 mg/day	Naïve	1	24 or 48	IDEAL study team	2009	RCT
Rumi et al. [9]	*α*-2a 180 ug/week; *α*-2b 1.5 ug/kg/week	800–1200 mg/day	Naïve	1,2,3,4	24 or 48	Italy	2010	RCT
Ascione et al. [10]	*α*-2a 180 ug/week; *α*-2b 1.5 ug/kg/week	1000–1200 mg/day	Naïve	1,2,3,4	24 or 48	Italy	2010	RCT
Mach et al. [11]	*α*-2a 180 ug/week; *α*-2b 1.5 ug/kg/week	1000–1200 mg/day	Naïve	1b	48	Poland	2011	RCT

**Table 2:** Baseline characteristics in the two groups of peginterferon *α*-2a and peginterferon *α*-2b in this meta-analysis.

Study	Peginterferon group	Total patients	Mean age (years)	Gender (male/female)	HCV genotype (1/2/3/4)	F3-4 OR cirrhosis, *N* (%)	Body weight (kg)	BMI (kg/m^2^)
Yenice et al. [5]	*α*-2a	37	49.95	13/24	37/0/0/0	NA	NA	NA
	*α*-2b	37	50.84	10/27	37/0/0/0	NA	NA	NA
Di Bisceglie et al. [6]	*α*-2a	189	46.9 ± 0.52	121/68	189/0/0/0	28 (14.8)	86.5 ± 1.34	29.2 ± 0.44
	*α*-2b	191	48.4 ± 0.56	136/55	191/0/0/0	29 (15.2)	85.4 ± 1.32	28.5 ± 0.42
Scotto et al. [7]	*α*-2a	71	45.86 ± 9.33	42/29	45/6/8/12	13 (18.3)	80.7	18.5–24.9 (*n* = 32), 25–29.9 (*n* = 34), ≥30 (*n* = 5)
	*α*-2b	72	47.82 ± 9.61	40/32	47/5/9/11	13 (18.1)	78.9	18.5–24.9 (*n* = 35), 25–29.9 (*n* = 30), ≥30 (*n* = 7)
McHutchison et al. [8]	*α*-2a	1035	47.6 ± 8.2	613/422	1035/0/0/0	110 (10.6)	82.8 ± 16.6	NA
	*α*-2b	1019	47.5 ± 7.8	613/406	1019/0/0/0	111 (10.9)	84.0 ± 16.5	NA
Rumi et al. [9]	*α*-2a	212	51.6 ± 12.0	128/84	91/69/34/18	43 (20.3)^†^	72.2 ± 14.6	25.5 ± 4.4
	*α*-2b	219	52.8 ± 12.0	120/99	87/74/32/26	39 (17.8)^†^	68.9 ± 12.0	24.8 ± 3.7
Ascione et al. [10]	*α*-2a	160	51.3 ± 10.3	81/79	89/49/18/4	33 (20.6)	70.4 ± 10.6	25.5 ± 3.1
	*α*-2b	160	48.9 ± 11.3	94/66	92/50/17/1	26 (16.3)	69.9 ± 10.7	25.3 ± 3.0
Mach et al. [11]	*α*-2a	138	45.2 ± 10.5	80/58	138/0/0/0	13 (9.4)	NA	24.5 ± 0.9
	*α*-2b	122	44.2 ± 13.6	73/49	122/0/0/0	12 (9.8)	NA	25.1 ± 1.3

NA: not available; BMI: Body mass index; ^†^Ishak score S5, 6.

F0–4 (F0: no fibrosis; F1: portal fibrosis without septa; F2: portal fibrosis with few septa; F3: numerous septa without cirrhosis; F4: cirrhosis).

All baseline characteristics were comparative between the two groups.

## Abbreviations

CHC: Chronic hepatitis C
HCV: Hepatitis C virus
RVR: Rapid virologic response
EVR: Early virologic response
ETR: End-of-treatment virologic response
SVR: Sustained virologic response
CI: Confidence interval.

## References

[1] Omata M, Kanda T, Yu ML (2012). APASL consensus statements and management algorithms for hepatitis C virus infection. *Hepatology International*.

[2] European Association for the Study of the Liver (2011). EASL clinical practice guidelines:management of hepatitis C virus infection. *Journal of Hepatology*.

[3] Seeff LB (2009). The history of the “natural history” of hepatitis C (1968-2009). *Liver International*.

[4] Schaefer EAK, Chung RT (2012). Anti-hepatitis C virus drugs in development. *Gastroenterology*.

[5] Yenice N, Mehtap O, Gümrah M, Arican N (2006). The efficacy of pegylated interferon alpha 2a or 2b plus ribavirin in chronic hepatitis C patients. *Turkish Journal of Gastroenterology*.

[6] Di Bisceglie AM, Ghalib RH, Hamzeh FM, Rustgi VK (2007). Early virologic response after peginterferon alpha-2a plus ribavirin or peginterferon alpha-2b plus ribavirin treatment in patients with chronic hepatitis C. *Journal of Viral Hepatitis*.

[7] Scotto G, Fazio V, Fornabaio C (2008). Peg-Interferon alpha-2a versus Peg-Interferon alpha-2b in nonresponders with HCV active chronic hepatitis: a pilot study. *Journal of Interferon and Cytokine Research*.

[8] McHutchison JG, Lawitz EJ, Shiffman ML (2009). Peginterferon alfa-2b or alfa-2a with ribavirin for treatment of hepatitis C infection. *The New England Journal of Medicine*.

[9] Rumi MG, Aghemo A, Prati GM (2010). Randomized study of peginterferon-*α*2a plus ribavirin vs peginterferon-*α*2b plus ribavirin in chronic hepatitis C. *Gastroenterology*.

[10] Ascione A, De Luca M, Tartaglione MT (2010). Peginterferon alfa-2a plus ribavirin is more effective than peginterferon alfa-2b plus ribavirin for treating chronic hepatitis C virus infection. *Gastroenterology*.

[11] Mach TH, Cieśla A, Warunek W (2011). Efficacy of pegylated interferon alfa-2a or alfa-2b in combination with ribavirin in the treatment of chronic hepatitis caused by hepatitis C virus genotype 1b. *Polskie Archiwum Medycyny Wewnetrznej*.

[12] Zhao SH, Liu EQ, Chen P (2010). A comparison of peginterferon *α*-2a and *α*-2b for treatment-naïve patients with chronic hepatitis C virus: a meta-analysis of randomized trials. *Clinical Therapeutics*.

[13] Awad T, Thorlund K, Hauser G, Stimac D, Mabrouk M, Gluud C (2010). Peginterferon alpha-2a is associated with higher sustained virological response than peginterferon alfa-2B in chronic hepatitis C: systematic review of randomized trials. *Hepatology*.

[14] Singal AK, Jampana SC, Anand BS (2011). Peginterferon alfa-2a is superior to peginterferon alfa-2b in the treatment of naïve patients with hepatitis C virus infection: meta-analysis of randomized controlled trials. *Digestive Diseases and Sciences*.

[15] Alavian SM, Behnava B, Tabatabaei SV (2010). The comparative efficacy and safety of peginterferon Alpha-2a vs. 2b for the treatment of chronic HCV infection: a meta-analysis. *Hepatitis Monthly*.

[16] Higgins JPT, Green S (2011). *Cochrane Handbook for Systematic Reviews of Interventions Version 5.1.0*.

[17] Higgins JPT, Thompson SG (2002). Quantifying heterogeneity in a meta-analysis. *Statistics in Medicine*.

[18] Berak H, Horban A, Wasilewski M (2005). Randomized, open label trial comparing efficacy and safety of pegylated interferon alfa 2a vs alfa 2b treatment of patients with chronic hepatitis C infected with non 2/3 genotypes—12 Week virological response analysis. *Hepatology*.

[19] Kolakowska-Rzadzko A, Berok H, Wasilewski M, Horbon A (2008). Relevance between fibrosis and response to treatment with peginterferon alfa 2a vs alfa 2b with ribavirin in chronic hepatitis C genotype 3 patients. Randomized open label study. *Hepatology*.

[20] Sinha S, Gulur P, Patel V, Hage-Nassar G, Tenner S (2004). A randomized prospective clinical trial comparing pegylated interferon alpha 2a/ribavirin versus pegylated interferon alpha 2b/ribavirin in the treatment of chronic hepatitis C. *The American Journal of Gastroenterology*.

[21] Khan AQ, Awan A, Shahbuddin S, Igbal Q (2007). Peginterferon alfa 2a/ribavirin versus peginterferon alfa 2b/ribavirin combination therapy in chronic hepatitis C genotype 3. *Gastroenterology*.

[22] Kamal S, Ghoraba D, Nabegh L (2009). Pegylated interferon alfa-2A vs. pegylated interferon alfa-2B, plus ribavirin for chronic hepatitis C genotype 4 patients: a randomized controlled trial. *Hepatology*.

[23] Magni C, Niero F, Argenteri B (2009). Antiviral activity and tolerability between pegylated interferon alpha 2a and alpha 2b in naive patients with chronic hepatitis C: results of a prospective monocentric randomized trial. *Hepatology*.

[24] Silva M, Poo J, Wagner F (2006). A randomised trial to compare the pharmacokinetic, pharmacodynamic, and antiviral effects of peginterferon alfa-2b and peginterferon alfa-2a in patients with chronic hepatitis C (COMPARE). *Journal of Hepatology*.

[25] Lee S, Kim IH, Kim SH (2010). Efficacy and tolerability of pegylated interferon-*α*2a plus ribavirin versus pegylated interferon-*α*2b plus ribavirin in treatment-naive chronic hepatitis C patients. *Intervirology*.

[26] Bruno R, Sacchi P, Ciappina V (2004). Viral dynamics and pharmacokinetics of peginterferon alpha-2a and peginterferon alpha-2b in naive patients with chronic hepatitis C: a randomized, controlled study. *Antiviral Therapy*.

[27] Laguno M, Cifuentes C, Murillas J (2009). Randomized trial comparing pegylated interferon -2b versus pegylated interferon -2a, both plus ribavirin, to treat chronic hepatitis C in human immunodeficiency virus patients. *Hepatology*.

[28] Scotto G, Fazio V, Fornabaio C (2008). Early and sustained virological response in non-responders with chronic hepatitis C: a randomized open-label study of pegylated interferon-*α*-2a versus pegylated interferon-*α*-2b. *Drugs*.

[29] Coppola N, Pisaturo M, Sagnelli C (2011). Efficacy and tolerability peginterferon a-2a and a-2b in patients with chronic hepatitis C by genotype 1: a meta-analysis. *Digestive and Liver Diseases*.

[30] Kau A, Vermehren J, Sarrazin C (2008). Treatment predictors of a sustained virologic response in hepatitis B and C. *Journal of Hepatology*.

[31] Ghany MG, Strader DB, Thomas DL, Seeff LB (2009). Diagnosis, management, and treatment of hepatitis C: an update. *Hepatology*.

[32] Fried MW, Shiffman ML, Reddy KR (2002). Peginterferon alfa-2a plus ribavirin for chronic hepatitis C virus infection. *The New England Journal of Medicine*.

[33] Hadziyannis SJ, Sette H, Morgan TR (2004). Peginterferon-alpha 2a and ribavirin combination therapy in chronic hepatitis C: a randomized study of treatment duration and ribavirin dose. *Annals of Internal Medicine*.

[34] Rumi M, Aghemo A, Prati GM (2012). Comparative trials of peginterferon *α*-2a and peginterferon *α*-2b for chronic hepatitis C. *Journal of Viral Hepatitis*.

[35] Hiramatsu N, Oze T, Yakushijin T (2009). Ribavirin dose reduction raises relapse rate dose-dependently in genotype 1 patients with hepatitis C responding to pegylated interferon alpha-2b plus ribavirin. *Journal of Viral Hepatitis*.

[36] Furusyo N, Murata M, Ogawa E (2011). Ribavirin concentration in the later stages of 48 week pegylated interferon-*α*2b plus ribavirin therapy for chronic hepatitis C is useful for predicting virological response. *Journal of Antimicrobial Chemotherapy*.

[37] Zeuzem S, Welsch C, Herrmann E (2003). Pharmacokinetics of peginterferons. *Seminars in Liver Disease*.

[38] Bruno R, Sacchi P, Scagnolari C (2007). Pharmacodynamics of peginterferon alfa-2a and peginterferon alfa-2b in interferon-naïve patients with chronic hepatitis C: a randomized, controlled study. *Alimentary Pharmacology and Therapeutics*.

[39] Foster GR (2010). Pegylated interferons for the treatment of chronic hepatitis C: pharmacological and clinical differences between peginterferon-*α*-2a and peginterferon-*α*-2b. *Drugs*.

[40] François C, Descamps V, Brochot E (2010). Relationship between the hepatitis C viral load and the serum interferon concentration during the first week of peginterferon-alpha-2b-ribavirin combination therapy. *Journal of Medical Virology*.

[41] Foster GR (2004). Review article: pegylated interferons: chemical and clinical differences. *Alimentary Pharmacology and Therapeutics*.

